# Effects of Ramadan Fasting on Recovery Following a Simulated Soccer Match in Professional Soccer Players: A Pilot Study

**DOI:** 10.3389/fphys.2019.01480

**Published:** 2019-12-06

**Authors:** Mohamed Amine Bouzid, Abd-Elbasset Abaïdia, Mustapha Bouchiba, Kais Ghattassi, Wael Daab, Florian A. Engel, Hamdi Chtourou

**Affiliations:** ^1^UR15JS01, Education, Motricité, Sport et Santé (EM2S), High Institute of Sport and Physical Education, University of Sfax, Sfax, Tunisia; ^2^Institute of Sport and Sport Science, Heidelberg University, Heidelberg, Germany; ^3^Activité Physique, Sport et Santé, UR18JS01, Observatoire National du Sport, Tunis, Tunisia; ^4^Institut Supérieur du Sport et de l’éducation physique de Sfax, University of Sfax, Sfax, Tunisia

**Keywords:** fast, sport, recovery, soccer, football

## Abstract

**Objectives:** Assessing the effects of Ramadan fasting on recovery following a soccer match simulation.

**Methods:** Eight elite soccer players (age: 21.0 ± 0.4 years) performed a modified Loughborough Intermittent Shuttle Test protocol (LIST_mod_) on two occasions: 1 week before (BR) and during the fourth week of Ramadan (End-R). At BR and End-R, soccer players performed squat jump, countermovement jump, maximal voluntary contraction, and 20 m sprint, and creatine kinase, uric acid, and subjective ratings (feelings scale, quality of sleep, fatigue, muscle soreness and stress) were assessed at baseline and 0, 24, 48, and 72 h following LIST_mod_.

**Results:** Following LIST_mod_, performance in squat jump (48 and 72 h) (*p* < 0.05), countermovement jump (48 and 72 h), maximal voluntary contraction (0, 24, 48, and 72 h), and 20 m sprint (0 and 48 h) decreased significantly on both occasions. Decreases were higher at End-R than BR. Creatine kinase levels increased significantly at 24 and 48 h at BR and End-R (*p* < 0.05). Uric acid increased at 0 and 24 h only on BR. Muscle soreness increased throughout the recovery period at both occasions, with a higher level at End-R. Stress rating increased only at 0 h on End-R, while fatigue rating increased at 24 h at BR and at 0, 24, and 48 h at End-R.

**Conclusion:** Perturbations in physical performance and subjective ratings parameters were higher at the end of Ramadan. However, the results of this study showed that Ramadan fasting did not adversely affect the recovery following soccer match simulation in professional soccer players.

## Introduction

A common challenge for Muslim soccer players is that competitions were scheduled during or shortly after Ramadan (e.g., Olympic Games in London in 2012, FIFA World Cup 2014 and 2018). During the 29–30 day period of Ramadan, observers are required to train or compete while refraining from drinking and eating from dawn to sunset (Zerguini et al., 2012). Given the constraints that Ramadan intermittent fasting (RIF) induces on Muslim soccer players, it has been demonstrated that RIF is associated with affecting exercise performance. Indeed, Zerguini et al. (2007) reported a decrease in running speed, agility, dribbling speed, and endurance performance in Algerian professional soccer players during Ramadan. Moreover, Chtourou et al. (2011) showed that performance during the shuttle run test, the Wingate Anaerobic Test as well as repeated speed ability were negatively affected by RIF in young soccer players.

Since observing Muslim athletes abstain from consumption of food and drinks during the hours of daylight, nutrient intake and drinking are restricted to the hours of darkness, which mostly results in a reduction of two meals per night (Shephard, 2012). In that context, a reduction of sleep duration is likely, since late evening meals and an early breakfast are common during Ramadan (Herrera, 2012). In addition, practical problems might occur with RIF in terms of a reduced availability of metabolites for both endurance and anaerobic exercise. In addition, cerebral consequences of a decreased blood glucose such as an increased perception of effort and a deterioration of mood state, a progressive decrease in hepatic and muscle glycogen reserves, a progressive fall of blood glucose levels and fluid reserves over the hours of daylight and risk of dehydration during prolonged effort may deteriorate exercise performance (Drust et al., 2012).

The above-mentioned alterations in drinking, sleep patterns, and glycogen reserves might probably impact the recovery following a competitive match in professional soccer players. In fact, the recovery process of fatigue mechanisms is highly variable and depends on several confounding factors such as the magnitude of fatigue induced by a soccer match, as well as extrinsic and intrinsic factors. In an analysis of the main factors affecting fatigue following a soccer match, Nédélec et al. (2012) reported that causes for fatigue are multifactorial but mainly related to glycogen depletion, sleep loss, and dehydration.

Studies describing mechanisms of post-match recovery in soccer are typically conducted over seasons of European or American leagues. To our knowledge, no study has focused on the post-match recovery of RIF soccer players during the period of Ramadan.

It is important to determine whether this religious fast has any untoward effects on the recovery process especially for Muslim soccer players because of their extensive participation, from recreational to professional standard, throughout the world and the heavy schedule of competitive soccer matches played in most leagues. With the help of this knowledge we are aiming for an optimization of the recovery period of observant soccer players. Therefore, the present study analyzed the post-match recovery period following a standardized exercise that replicates the fatigue to a soccer match during the month of Ramadan in professional soccer players. We speculate that RIF would attenuate the recovery process following a simulated soccer match.

## Materials and Methods

### Participants

Eight professional soccer players (age: 21.0 ± 0.4 years; body mass: 71.97 ± 2.52 kg; height: 176.7 ± 3.1 cm) from a Tunisian club volunteered to participate in this study. The club competed in the first division of Tunisian National Junior League. After receiving a description of the study protocol, including the possible risks and benefits associated with the study, each subject signed a written informed consent prior to participation. The study was conducted in accordance with the Code of Ethics for human experimentation, the Declaration of Helsinki (World Medical Association, 2013) and was approved by the research ethics committee of Habib Bourguiba University Hospital, Sfax, Tunisia (CCP N°: 16/04). All participants observed the traditional Ramadan fasting, abstaining from any food and fluid intake from dawn to sunset. All participants trained at least 4 days per week with an average of 1 h and 30 min per day. During the study, participants were regularly exercising to maintain their physical performance and undertaking their usual training sessions supervised by their coaches. Training sessions were scheduled from 16:30 to 18:30 h during control period, and after breaking the fast during Ramadan (i.e., from 21:30 to 23:30 h). In both periods, training sessions lasted for ~60 min: 15 min warm-up, 15 min conditioning training, 30 min technical and tactical skill training. All participants were practicing Muslims and did fast from sunrise to sunset during Ramadan.

### Experimental Design

**Figure 1:**
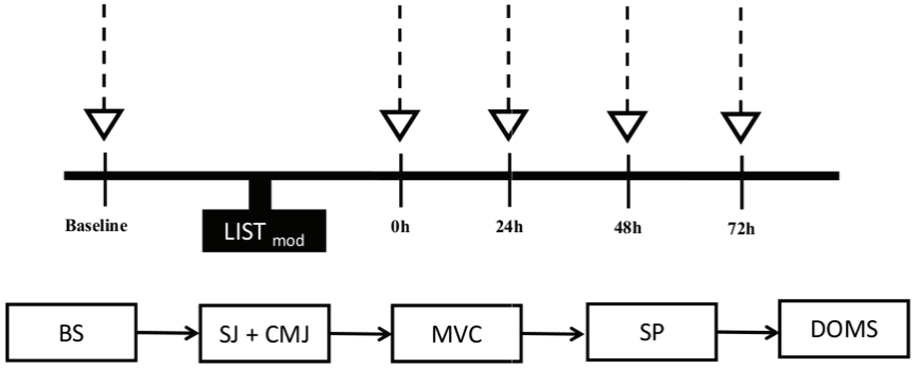
Experimental design. Downward arrows denote the time points when physical performance tests, blood samples, and perceptual ratings were recorded 1 week before (BR) and during the fourth week of Ramadan (End-R). Blood sample (BS), squat jump (SJ), countermovement jump (CMJ), maximal voluntary contraction of quadriceps (MVC), and 20 m sprint test (SP).

The study was conducted in Sfax, Tunisia, in 2016. Ramadan started on the 6th of June and ended on the 5th of July. The length of each day-time fast was approximately 16 h starting at ~03:30 h and ending at ~19:30 h. The participants were first asked to visit the laboratory 14 days before Ramadan to familiarize themselves with all involved testing procedures of the study. Participants completed an initial session to ensure familiarity with all measures and procedures, followed by two experimental testing sessions. The first experimental session was performed 1 week before Ramadan (BR) and the second experimental session was performed at the end of the last week of Ramadan (Figure 1) (End-R). In each experimental session, participants performed a modified Loughborough Intermittent Shuttle Test protocol (LIST_mod_) (Stone and Oliver, 2009) at the same time of day (between 16:00 and 18:00 h) to minimize diurnal variation effects. Physical, biochemical, and perceptual markers were obtained at baseline, 0, 24, 48, and 72 h after the LIST_mod_. During the period devoted to each condition, no training session was implemented and participants were requested to refrain from any specific recovery treatment.

### Soccer Match Simulation

In each experimental session, participants performed a 45 min intermittent running protocol (LIST_mod_), consisting of three 15-min blocks interspersed by a 3 min passive recovery period. The Intermittent running test was designed by Stone and Oliver (2009) and is a modification of the Loughborough Intermittent Shuttle Test protocol (Nicholas et al., 2000). LIST_mod_ aims to replicate a competitive soccer match and is suggested to induce the fatigue responses to a soccer match because it includes multiple frequent accelerations and decelerations as well as high-intensive bouts inherent in soccer match play (Buchheit et al., 2010). During the LIST_mod_, players run at various speeds covering walking, easy jogging, running as well as sprinting between two lines, 20 m apart. The walking and running speeds during each 20 m shuttle of the test were dictated by verbal instructions and audible beeps prerecorded on compact disc. To assist with pacing, different pitch audible beeps are signaled (Stone and Oliver, 2009). Before the intermittent running test, a warm-up session was performed by players on the same pitch as the LIST_mod_. The warm-up session was identical with the team’s standardized pre-match warm-up protocol and included light jogging, dynamic activities (buttock kicks, high knee lifts, backward running, side-steps), short accelerations, and sprints as well as movement preparation exercises for a total duration of 15 min. Heart rate was continuously recorded throughout LIST_mod_ with electrode chest belt and the Polar Team System (Polarelectro, Kempele, Finland).

### Physical Performance Tests

For each session, the order of physical performance tests was identical throughout the protocol: squat jump (SJ), countermovement jump (CMJ), maximal voluntary contraction of quadriceps (MVC), and 20 m sprint (SP).

Initially, participants performed the same standardized 15-min warm-up comprising easy jogging, 3–5 accelerations, movement flow consisting of body weight squat variations, lunges, and hip mobility drills.

Each athlete performed three SJs and three CMJs using Opto-jump system (Microgate SARL, Italy), the best jump height of SJ and CMJ was recorded for analysis. During SJ and CMJ, players placed their hands on the hips.

Subsequently, participants performed brief isometric maximal voluntary contractions (MVCs) of knee extension with 90° flexion on an isometric dynamometer (Good Strength, Metitur, Finland). Before MVC, participants performed specific warm-up consisting of three sub-maximal repetitions of knee extension, starting from 90° flexion. For MVC, players completed two maximal repetitions of knee extension with knee flexed at 90°; between the two trials, participants rested for 60 s. During testing, participants were seated on an isometric dynamometer (Good Strength, Metitur, Finland) and participants were stabilized with safety belts strapped across the chest, thighs, and hips, in order to avoid lateral, vertical, or frontal displacements. All measurements were taken from the participant’s dominant leg, with 90° knee flexion angle from full extension.

Following MVC test, participants performed the 20 m sprint test. For determining sprint performance, we used single beam timing lights (Brower Timing System, IRD-T175, Draper, UT, USA) placed at 0 and 20 m. The two timing lights (start and finish) were mounted at 100 cm, in order to approximately match hip height. Players were asked to start each sprint from a line placed 50 cm before the starting line. Participants began running upon a verbal signal and they were instructed to run as fast as possible to complete the 20-m distance. Players performed one familiarization sprint followed by 3 min of recovery. Subsequently, players performed two trials interceded by 3 min of recovery, the best trial was recorded for subsequent analysis.

### Measurements

#### Blood Analysis

During each experimental session and at each time point, ~5 ml of venous blood was taken from an antecubital vein into a plain Vacutainer tube. Whole blood collected into tubes containing no additive was allowed to clot for 30 min at room temperature, centrifuged at 1,500 rpm for 15 min at 4°C, and then processed for plasma and stored at −70°C until analysis. Plasma creatine kinase activity (CK) and the concentrations of uric acid (UA) were determined spectrophotometrically using commercial test kits (A11A01632, Horiba-ABX, Montpellier, France; myoglobin bioMerieux 30446 and Roche Diagnostics, Carnaxide, Portugal respectively) and according to the instructions of the manufacturers. To avoid variations in assay conditions, each assay was conducted in duplicate on the same day, and within 7 days of blood collection. The inter- and intra-assay coefficients of variation for CK and UA were 2.1–4.3 and 1.6–5.1, respectively.

#### Perceptual Scales

Following the LIST_mod_, participants rated the global session intensity with the Borg scale (Haddad et al., 2013) and rated perceived feelings during LIST_mod_ using the feelings scale (Rejeski et al., 1987). The RPE scale allows athletes to give a subjective exertion rating for the physical exercise. It consists of a 11-point scale ranging from 0 (very very light) to 10 (very very hard). The feelings items were rated on an 11-point scale: +5, very good; +3, good; +1, fairly good; 0, neutral; −1, fairly bad; −3, bad; and −5, very bad.

Each morning at the same time (i.e., 24, 48, and 72 h following the LIST_mod_), players rated their quality of sleep, fatigue, muscle soreness, and stress (Hooper et al., 1995). This method allows one to follow four variables: perceived sleep quality; perceived quantities of stress; delayed onset muscle soreness (DOMS); and general fatigue using a scale from 1 to 7 points.

#### Body Composition

For all participants, body height was measured using a stadiometer and body weight was measured using a calibrated electronic scale (Tanita TBF 401, Tanita Corp., Japan). Total body water and body fat percentage were assessed by using bioelectrical impedance analyzer (Tanita TBF 401, Tanita Corp., Japan). Finally, in order to determine sweat loss during the LIST_mod_, players were weighed wearing dry shorts before and immediately after the exercise using a digital scale (Tanita TBF 401, Tanita Corp., Japan).

#### Dietary Intake Analysis

Participants recorded all food and fluids intake during the week before Ramadan and then 3 days per week during Ramadan. Dietary records were analyzed using the Bilnut program (Nutrisoft, Cerelles, France) and the food-composition tables of the National Institute of Statistics of Tunis in 1978. Total water intake was defined as the fluid volume of consumed beverages plus the water content of consumed foods.

#### Statistical Analyses

All data are presented as means ± standard deviation (SD). Data were analyzed using Statistica for Windows software (version 6.0, StatSoft, Inc., Tulsa, OK). Values for physical and biochemical parameters were normalized to 100%. Once the assumption of normality was confirmed using the Shapiro-Wilk W-test, parametric tests were performed. Anthropometric measures and dietary data between BR and End-R periods were analyzed using paired Student’s t test. For all physiological, biochemical, and perceptual data, a two-way ANOVA with repeated measures [Sessions (BR or End-R) × time (Baseline, 0, 24, 48, and 72 h post exercise)] was performed. When appropriate, *post hoc* comparisons were used with the Bonferroni test. Power calculation was made using G*Power v.3 (Faul et al., 2009). Effect sizes (ESs) were calculated to determine the magnitude and practical relevance of changes [<0.2 = trivial, 0.2–0.6 = small, 0.6–1.2 = moderate, 1.2–2.0 = large, and >2.0 = very large (Hopkins, 2002)]. For reliability of biochemical and physiological data, intraclass correlation coefficient (ICC), the coefficient of variation (CV), typical error (TE), and 90% confidence intervals (CIs) were calculated. Statistical significance was accepted at *p* < 0.05.

## Results

### Body Weight, Body Composition, Dietary Intake, and Physiological Responses

As shown in Table 1, total body weight and total body water were lower during End-R compared to BR (−4.5% respectively −2.9%) (*p* < 0.05). However, body fat percentage and body mass index did not change between the two periods (*p* = 0.12).

**Table 1:** Descriptive characteristics (mean ± SD) of participants recorded before Ramadan and at the end of Ramadan.

	Before Ramadan	End of Ramadan
Age (years)	21.0 ± 0.4	—
Weight (kg)	71.97 ± 2.52	68.16 ± 3.33^*^
Height (cm)	176.7 ± 3.1	—
Body mass index (kg/m^2^)	23.23 ± 1.4	22.01 ± 1.1
Body fat (%)	9.79 ± 2.45	8.92 ± 2.98
Total body water (%)	63.29 ± 1.92	61.56 ± 1.64^*^
Training sessions h/week	6.1 ± 0.3	5.8 ± 0.5

* *Significantly different from before Ramadan period (p < 0.05)*.

The values of estimated daily dietary intake during BR and at End-R are reported in Table 2. Comparison of daily mean energy and macro-nutrient intakes by the participants between the two periods showed no significant statistical differences (*p* = 0.07). However, daily water intake was significantly higher during BR compared to End-R (*p* < 0.05). Fluid loss during the intermittent exercise was 0.89 ± 0.18 l and 0.92 ± 0.3 l (or 1.2 ± 0.4% and 1.3 ± 0.3% of body mass) for BR and End-R respectively (*p* = 0.06). No significant differences were observed between the mean heart rate during the test on BR (151 ± 15 bpm) and End-R (145 ± 14 bpm).

**Table 2:** Dietary intake (mean ± SD) recorded before and at the end of Ramadan.

	Before Ramadan	End of Ramadan
Energy intake (kcal/day)	2075 ± 645.87	2238.75 ± 578.43
Carbohydrate (g/day)	254.38 ± 79.8	290.63 ± 56.85
Carbohydrate (%)	49.88 ± 6.94	50.88 ± 6.45
Protein (g/day)	80 ± 36.55	78.75 ± 18.85
Protein (%)	15.25 ± 3.49	14.2 ± 3.63
Fat (g/day)	70.9 ± 27.4	69.5 ± 16.3
Fat (%)	30.76 ± 3.4	29.8 ± 4.2
Total water intake (L/day)	4.0 ± 0.5	3.3 ± 0.2^*^

* *Significantly different from before Ramadan (p < 0.05)*.

### Reliability

The reliability of each outcome measure was analyzed using the results of baseline test of BR and End-R (Table 3). Coefficient of variation, intraclass correlation coefficient (ICC), and typical error are criteria for reliability (Vincent and Weir, 1999). Reliability is generally considered high when ICC is above 0.90 and good when ICC is above 0.75 (Cohen, 1988). Reliability for CK (ICC = 0.72), UA (ICC = 0.63), and SP (ICC = 0.69) was low. However, a good level of reliability was reported for MVC (ICC = 0.79), CMJ (ICC = 0.88), and SJ (ICC = 0.77). Typical error was low for all the tests, which indicates a good reliability – as the smaller the typical error the more reliable the measurements. A coefficient of variation is generally considered high when it is above 10% (Atkinson and Nevill, 1998). For SP (CV = 11.2%), coefficient of variation was high. For MVC (CV = 8.8%), CMJ (CV = 9.4%), and SJ (CV = 9.2%) coefficients of variation were low.

**Table 3:** Reliability of outcomes measured during the experimental protocol.

	Trial 1	Trial 2	Effect size (90% CI)	TE (90% CI)	ICC (90% CI)	CV
SJ (cm)	39.5 ± 4.1	35.8 ± 5	0.81 (90% CI = −0.09 to 1.62)	2.51 (90% CI = −1.77 to 4.50)	0.78 (90% CI = 0.20 to 0.91)	9.6%
CMJ (cm)	39.2 ± 4.3	37.5 ± 3.8	0.39 (90% CI = −0.46 to 1.20)	1.0 (90% CI = −0.05 to 2.39)	0.80 (90% CI = 0.3 to 1)	9.5%
MVC (N)	733 ± 119.1	823.8 ± 101.4	0.14 (90% CI = −0.2 to 0.09)	6.2 (90% CI = −12.1 to 31. 9)	0.75 (90% CI = 0.03 to 0.98)	8.6%
SP (s)	3.1 ± 0.1	3.2 ± 0.2	−0.63 (90% CI = −1.44 to 0.24)	0.16 (90% CI = −0.11 to 0.28)	0.67 (90% CI = −0.1 to 0.81)	12.2%

*Trial 1 and trial 2 are respectively the baseline values of the two conditions. CV, coefficient of variation. ICC, intraclass correlation coefficient; CI, 90% confidence intervals; TE, typical error*.

*SJ, Squat jump; CMJ, countermovement jump; MVC, maximal voluntary contraction; SP, 20 m sprint test*.

### Recovery for Physical Performance

**Figure 2:**
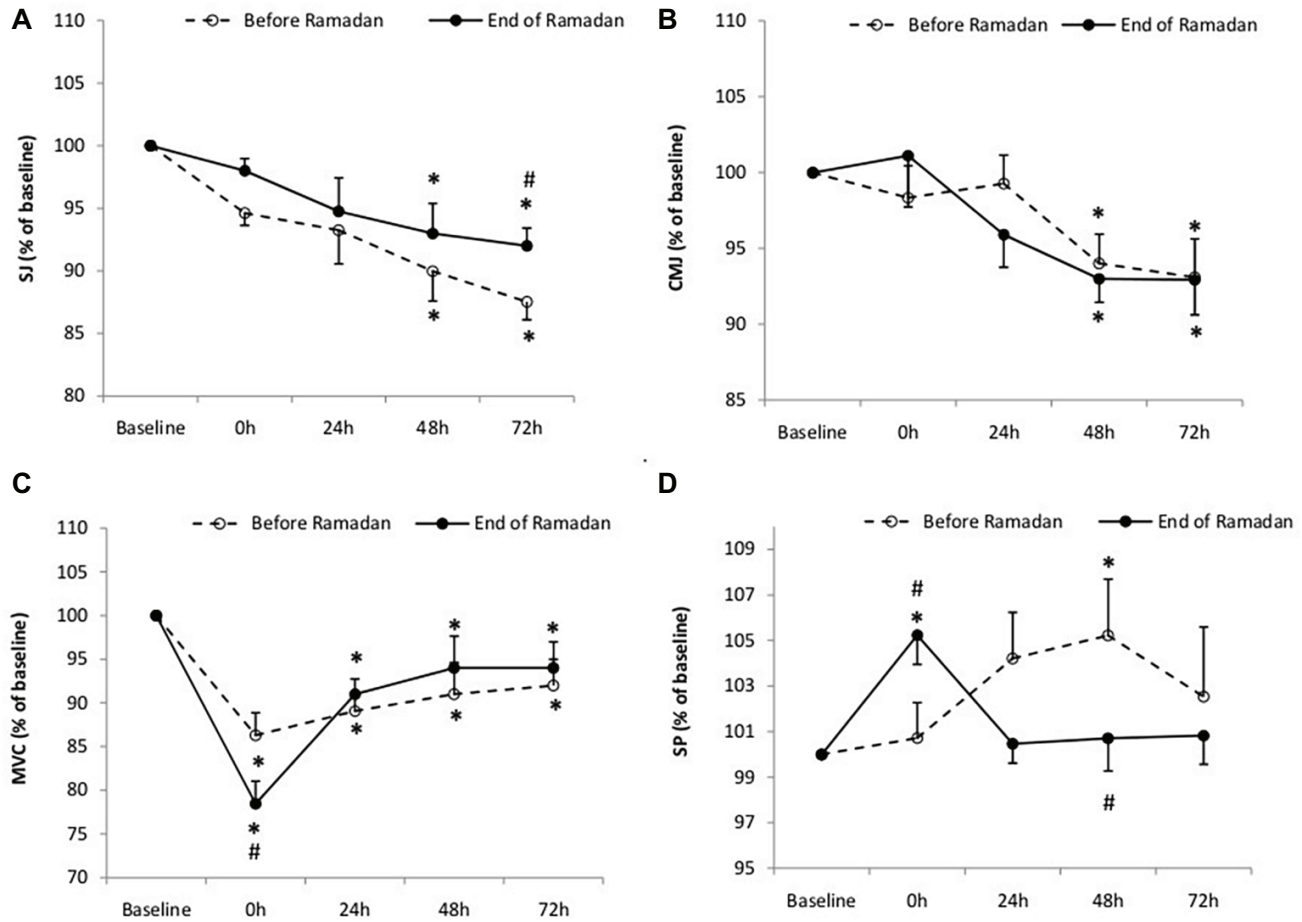
Squat jump (SJ) **(A)**, countermovement jump (CMJ) **(B)**, maximal voluntary contraction of quadriceps (MVC) **(C)**, and 20 m sprint test (SP) **(D)** values recorded at baseline and following the intermittent test (at 0, 24, 48, and 72 h) before and at the end of Ramadan. Values are means and standard deviations. ^*^Significant difference in comparison to baseline (*p* < 0.05). ^#^Significant difference in comparison to before Ramadan (*p* < 0.05).

Statistical analysis showed an interaction between conditions and measurement time for height in the SJ (*F*_4,28_ = 5.99, *p* = 0.04, ES = 0.44) and an effect of measurement time for CMJ performance (*F*_4,28_ = 4.59, *p* = 0.001, ES = 0.53). During BR and End-R, *post hoc* analysis showed a significant decrease in SJ at 48 h (*p* = 0.03; *d* = 0.58; 95%CI = −0.29, 1.38 and *p* = 0.02; *d* = 0.68; 95%CI = −0.19, 1.48, respectively) and 72 h (*p* = 0.04; *d* = 0.64; 95%CI = −0.24, 1.44 and *p* = 0.001; *d* = 0.67; 95%CI = −0.21, 1.48, respectively) (Figure 2A). However, this decrease in SJ was significantly lower in End-R as compared to BR (*p* = 0.01; *d* = 0.77; 95%CI = −0.12, 1.58). Likewise, CMJ height decreased at 48 and 72 h during BR (*p* = 0.03; *d* = 0.57; 95%CI = −0.30, 1.38 and *p* = 0.01; *d* = 1.72; 95%CI = 0.68, 2.57, respectively) and End-R (*p* = 0.04; *d* = 0.88; 95%CI = −0.03, 1.69 and *p* = 0.02; *d* = 0.42; 95%CI = −0.42, 1.24, respectively), but without any significant difference between the two periods throughout the recovery period (Figure 2B).

On the other hand, we observed a significant interaction effect for MVC (*F*_4,28_ = 6.38, *p* = 0.04, ES = 0.49) (Figure 2C). *Post hoc* analysis showed that, during BR and End-R, MVC performance decreased following LIST_mod_ at 0 h (*p* = 0.01; *d* = 1.72; 95%CI = 0.68, 2.57 and *p* = 0.04; *d* = 1.22; 95%CI = 0.58, 1.95), 24 h (*p* = 0.01; *d* = −0.12; 95%CI = −0.29, 1.08 and *p* = 0.04; *d* = 0.83; 95%CI = 0.22, 1.54), 48 h (*p* = 0.001; *d* = 0.79; 95%CI = −0.49, 1.51 and *p* = 0.03; *d* = 0.58; 95%CI = −0.29, 1.38), and 72 h (*p* = 0.01; *d* = −1.03; 95%CI = −0.11, −1.85 and *p* = 0.03; *d* = 0.42; 95%CI = −0.44, 1.22). However, MVC performance decrement following LIST_mod_ (at 0 h) was significantly higher in End-R compared to BR (*p* = 0.01; *d* = 0.78; 95%CI = 0.19, 1.78).

Furthermore, for SP performance, an effect of interaction between condition and time was observed (*F*_4,28_ = 5.14, *p* = 0.03, ES = 0.32) (Figure 2D). SP decreased following LIST_mod_ (0 h) (*p* = 0.02; *d* = 0.42; 95%CI = −0.42, 1.24) and returned to baseline levels 24 h following LIST_mod_ in End-R. However, SP decreased only at 48 h (*p* = 0.01; *d* = 1.04; 95%CI = 0.11, 1.85) and returned to baseline levels 72 h after LIST_mod_ in End-R (Figure 2C). Moreover, SP performance was significantly lower at 0 h (*p* = 0.04; *d* = 0.6; 95%CI = −0.01, 1.15) and was significantly higher at 48 h following LIST_mod_ (*p* = 0.001; *d* = 0.38; 95%CI = −0.09, 1.78) in End-R compared to BR.

### Recovery for Biochemical Parameters

**Figure 3:**
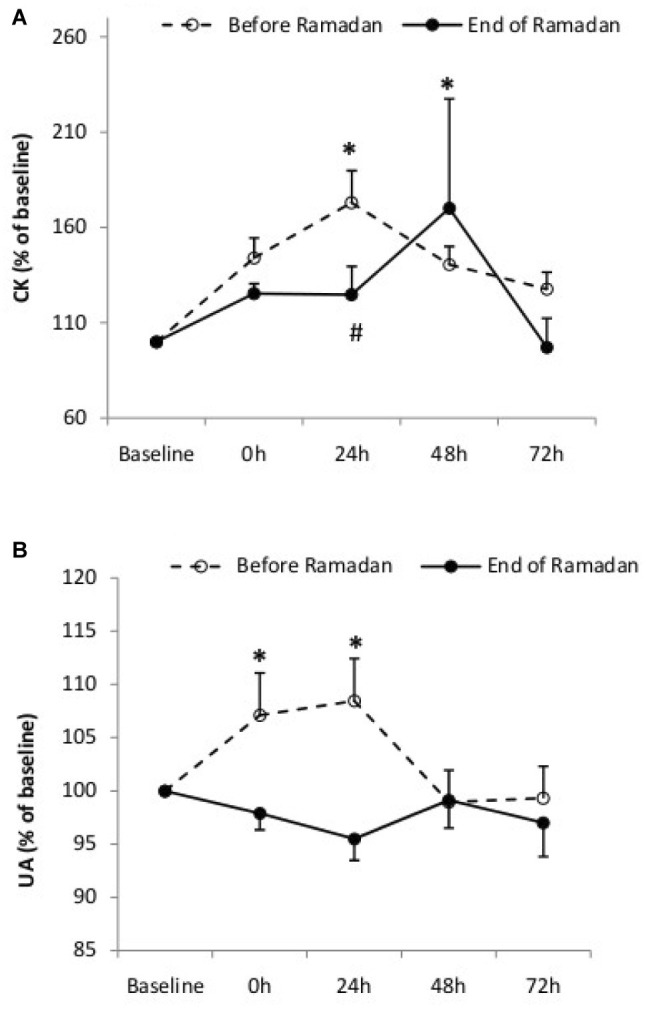
Plasma creatine kinase (CK) activity **(A)** and uric acid (UA) level **(B)** recorded at baseline and following the intermittent test (at 0, 24, 48, and 72 h) before and at the end of Ramadan. Values are means and standard deviations. ^*^Significant difference in comparison to baseline (*p* < 0.05).

Statistical analysis showed a significant interaction between condition and time for CK (*F*_4,28_ = 7.52, *p* = 0.03, ES = 0.69). As shown in Figure 3, CK activity increased only at 0 h during BR (*p* = 0.01; *d* = −1.31; 95%CI = −2.14, −0.34) and only at 48 h in End-R (*p* = 0.01; *d* = 0.72; 95%CI = −0.13, 1.57) (Figure 3A). In addition, CK level was significantly lower at 24 h in End-R compared to BR (*p* = 0.001; *d* = 0.44; 95%CI = −0.11, 1.91). Furthermore, UA concentration peaked at 0 h (*p* = 0.01; *d* = 0.6; 95%CI = −0.1, 1.22) and 24 h (*p* = 0.01; *d* = 0.51; 95%CI = −0.14, 1.29) after LIST_mod_ and returned to baseline levels 48 h following LIST_mod_ in BR (Figure 3B). However, no significant changes in UA concentration were observed immediately after or throughout the 72 h following LIST_mod_ during End-R (*p* = 0.13; *d* = 4.47; 95%CI = 2.75, 5.71).

### Recovery for Subjective Ratings

RPE and the perceptual feelings scores at the end of LIST_mod_ were 8.5 ± 0.3 and −0.3 ± 1.3 during BR respectively and 7.8 ± 0.2 and −0.4 ± 2.4 during End-R, respectively. No significant difference was found in RPE and feelings scores between the two periods (*p* = 0.09). Quality of sleep, fatigue, muscle soreness, and stress before the LIST_mod_ and throughout the recovery period during BR and End-R periods are reported in Table 4. Neither Ramadan fasting nor the LIST_mod_ affected the quality of sleep of participants (*p* = 0.13). Fatigue rating increased at 24 h in BR (*p* = 0.01; *d* = 0.42; 95%CI = −0.42, 1.24) and at 0 h; (*p* = 0.02; *d* = 0.33; 95%CI = −0.30, 1.38); 24 h (*p* = 0.03; *d* = 0.58; 95%CI = −0.29, 1.38); and 48 h (*p* = 0.03; *d* = 0.47; 95%CI = −0.3, 1.48) in End-R. In addition, fatigue rating was significantly higher at 0 h (*p* = 0.01; *d* = 0.72; 95%CI = −0.13, 1.57) and 48 h (*p* = 0.04; *d* = 0.6; 95%CI = −0.01, 1.15) in End-R as compared to BR. Significant increases in stress rating were observed only at 0 h in End-R. Moreover, muscle soreness rating increased throughout the recovery in both periods (*p* = 0.01; *d* = 0.77; 95%CI = −0.12, 1.58). However, this increase immediately after LIST_mod_ at 0 h (*p* = 0.02; *d* = 0.67; 95%CI = −0.21, 1.48), 24 h (*p* = 0.01; *d* = 0.90; 95%CI = −0.01, 1.70), and 48 h (*p* = 0.01; *d* = 0.64; 95%CI = −0.24, 1.44) was significantly higher in End-R as compared to BR.

**Table 4:** Subjective ratings scores (mean ± SD) recorded before and at the end of Ramadan at baseline immediately after 0, 24, 48, and 72 h after the simulated soccer match.

	Before Ramadan					End of Ramadan				
	Baseline	0 h	24 h	48 h	72 h	Baseline	0 h	24 h	48 h	72 h
Sleep (au)	4.43 ± 1.27	—	3.57 ± 0.98	4.29 ± 0.76	3.75 ± 1.49	3.9 ± 1.1	—	4.0 ± 0.82	3.75 ± 0.5	4.29 ± 1.11
Fatigue (au)	2.3 ± 1.3	2.4 ± 1.7	3.43 ± 0.79^*^	2.9 ± 0.82	3.25 ± 1.04	3.02 ± 1.11	4.7 ± 1.30^*^^,^^#^	3.99 ± 0.49^*^	4.29 ± 0.95^*^^,^^#^	3.2 ± 0.82
Stress (au)	1.9 ± 1.1	2.1 ± 0.9	2.86 ± 1.57	2.5 ± 1	2.38 ± 1.06	2.29 ± 1.25	4.0 ± 0.8^*^^,^^#^	2.14 ± 0.90	3.0 ± 0.82	3.0 ± 0.58
Muscle soreness (au)	1.2 ± 0.9	2.7 ± 1.1^*^	2.8 ± 1.07^*^	3.1 ± 1^*^	2.63 ± 0.74^*^	1.17 ± 1.13	3.6 ± 0.45^*^^,^^#^	3.98 ± 1.21^*^^,^^#^	4.14 ± 0.69^*^^,^^#^	3.29 ± 0.95^*^

*au, arbitrary unit*.

* *Significantly different from baseline (p < 0.05)*.

# *Significantly different from before Ramadan (p < 0.05)*.

## Discussion

The aim of this study was to analyze the recovery following a simulated soccer match (LIST_mod_) in elite soccer players during two different conditions: (1) 1 week before (BR) and (2) during the fourth week of Ramadan (End-R). The main findings of this study were that the recovery of physical performance markers was similar between BR and End-R period. In addition, biochemical responses to the LIST_mod_ throughout the recovery period seem to be unaffected by Ramadan fasting. Perceived stress, fatigue, and muscle soreness following the exercise were higher during End-R, while no difference was observed between BR and End-R in quality of sleep.

The present findings demonstrate that the daily estimated total energy intake was not affected by Ramadan fasting. In line with the present results, previous research reported that the total daily energy intake did not differ between BR and during Ramadan in healthy Tunisian participants (El Ati et al., 1995; Beltaifa et al., 2002; Alsubheen et al., 2017). Yet, other studies reported a significant decrease (Bouhlel et al., 2006), or a significant increase (Frost and Pirani, 1987) of Ramadan fasting on daily energy intake. The differences in daily energy intake between the studies could likely be due to nutritional customs and personal habits as well as social conditions and geographical differences of study participants.

Exercise performance parameters are frequently utilized as reliable indicators for analyzing recovery of physical performance (Warren et al., 1999). Consistent with previous studies, the soccer match simulation was effective to induce a pronounced decrease in neuromuscular performance (Andersson et al., 2008; Ascensão et al., 2008; Magalhães et al., 2010). Most studies related to Ramadan fasting and physical exercise focus on comparing physical performance during and following Ramadan. Some studies showed that Ramadan fasting decreases physical performance (Meckel et al., 2008; Chennaoui et al., 2009), while others did not reveal any effects of Ramadan fasting (Kirkendall et al., 2008; Zerguini et al., 2008; Memari et al., 2011; Chtourou et al., 2012; Hammouda et al., 2013). Results of the present study demonstrated that physical performance before and at the end of Ramadan needed between 24 and 72 h of recovery to return to baseline levels. In addition, recovery of physical performances was similar between BR and End-R periods, but with more pronounced responses to LIST_mod_ during Ramadan fasting. Therefore, several possible factors have to be considered to explain the pronounced decrease in physical performance throughout the recovery period during End-R.

During End-R, participants had approximately 12 h between the last meal and the LIST_mod_ in the present study. It is likely that the 12 h of fasting potentially cause dehydration as well as other metabolic and hormonal alterations (El Ati et al., 1995) that show an impact on exercise performance (i.e., muscle power and fatigue) during the recovery period.

In addition, studies demonstrated a potential decrease in sleep duration during Ramadan in elite athletes (Waterhouse et al., 2009; Herrera, 2012). During Ramadan, individuals tend to wake up earlier and eat a meal before sunrise, to prepare for the period of fasting. As a result, participants could suffer from partial sleep deprivation, which could have a negative impact on mood, mental performance, as well as on physical activity during subsequent days (Reilly and Edwards, 2007).

On the other hand, concentrations of both plasma CK and UA are indicators to quantify muscle membrane disruption and purine metabolism because CK diffuses into the plasma from damaged skeletal muscle fibers. The results of the present study showed that recovery of CK activity was similar between BR and End-R periods. Indeed, in both periods, CK activity peaked at 24 h and returned to baseline values 48 h after the LIST_mod_. The results of the present study clearly demonstrate that Ramadan fasting did not adversely affect the recovery of cellular damage after a simulated soccer match.

Regarding UA levels, the present study demonstrated that UA concentration increased at 0 and 24 h following the simulated soccer match and returned to baseline levels 48 h after the simulated match in BR, but without any significant changes during End-R. Majority of studies, focusing on UA and exercise during the month of Ramadan reported that resting and post-exercise levels of UA increased significantly during Ramadan (Chaouachi et al., 2008; Hammouda et al., 2014). These authors refer the increased UA concentrations observed during Ramadan to dehydration and an excessive rate of protein breakdown. In the present study, the mechanism(s) responsible for the lower exercise-induced UA level during the recovery period in End-R remains unclear. Previous research demonstrated that UA represents ~60% of the antioxidant capacity of plasma, and its protective role against reactive oxygen species generation during exercise was emphasized (Ascensão et al., 2008; Ispirlidis et al., 2008). On the other hand, several studies reported that Ramadan fasting enhances antioxidant defenses in athletes (Trabelsi et al., 2011; Hammouda et al., 2014). Accordingly, the lack of changes of UA level during the recovery in End-R observed in the present study could be explained by the protective role of other antioxidants, like vitamin E and β-carotene, which could be enhanced by Ramadan fasting.

No difference was observed between BR and End-R for the reported quality of sleep. The absence of alterations in Hooper’s Index does not necessarily mean that sleep was not impacted by Ramadan fasting, since the Hooper’s Index is a simple general index assessing the perceived sleep quality (Chamari et al., 2012). Even if participants were satisfied with their 24-h sleep quality, time spent in the different sleeping phases could potentially be modified. Moreover, perceived stress, assessed by the Hooper Index, seems to be higher in End-R. This result is not in line with Chamari et al. (2012), who reported a lower stress level on fasting soccer players during the month of Ramadan. The authors speculate that Ramadan fasting potentially caused a lower perception of stress in the fasting players since Ramadan observance matches the religious beliefs and therefore increases the perceived well-being of religious players. Whereas, in the present study, an increase in perceived fatigue and muscle soreness throughout the recovery period with Ramadan fasting was observed. This result is in accordance with recent studies of Chamari et al. (2016) and Chtourou et al. (2012) showing increased fatigue at End-R. In the present study, the exact underlying mechanisms for the increase in perceived fatigue and muscle soreness in the recovery period during Ramadan remain unclear. A potential explanation could be that training load increased during the month of Ramadan. In the present study, training load was estimated based on the RPE method, and no significant difference in training load between BR and End-R was observed. However, even if the RPE method has been validated and used in soccer players to quantify training load, it is likely that training load increased progressively, but still non-significantly, during Ramadan, resulting in a slight increase in external training load, leading to an increase in perceived fatigue and muscle soreness.

Some limitations inherent to the experimental protocol of this study warrant mention. First, the statistical power is very low (around 40%) due to the small sample size in the present study. However, this limitation is tempered by the use of professional players in our study. It was deemed necessary to recruit professional soccer players as the LIST_mod_ was based on data collected on top-level soccer players. Another major limitation of the present study is the absence of a control group (e.g., soccer players not fasting during Ramadan). But it should be noted that obtaining non-fasting participants in countries with a Muslim majority is not feasible due to ethical and practical reasons. Therefore, the majority of studies on Ramadan fasting conducted in Muslim countries used pre-Ramadan values as control/baseline condition. Moreover, it would be interesting if the first data collection would be timed 1 week following the beginning of Ramadan, since Ramadan-induced alterations are potentially attenuated in the beginning of Ramadan compared to the end of Ramadan. Future studies investigating recovery in athletes during Ramadan period should collect data before, at the beginning, and at the end of Ramadan period in order to reveal any effects of Ramadan fasting. In addition, the main differences in the present study were found in self-reported parameters. In order to make the evaluation of self-reported parameters more objective, examiners provided the standardized instructions for the use of every scale to participants and participants were encouraged to give the maximum objective score that reflect their real feeling independently of their belief about Ramadan. Therefore, in the present study, we are confident that all participants satisfactorily understood the use of the perceived feelings scale questionnaire. Finally, we would use gravity or osmolarity technique in order to be more objective to evaluate hydration status. Unfortunately, for technical reasons, we were unable to use these methods.

## Conclusions

In conclusion, this pilot study conducted in professional soccer players showed that Ramadan fasting does not adversely affect the recovery of physical performance and muscle damage markers, while perceived fatigue and muscle soreness seems to be enhanced with Ramadan fasting. These findings may indicate that exercising in the Ramadan fasted state may not additionally increase the risk of unfunctional overreaching or overtraining for athletes, as their capacity of recovery is maintained during the month of Ramadan. Contrary to the existing literature, we suggest that when planning training sessions for elite soccer players during Ramadan, coaches could keep the same training volume and intensities without a significant increased risk of overreaching or overtraining. Finally, further experimental investigations are required to confirm the present study findings in order to broaden the knowledge about the perturbations involved with training and recovery in elite athletes during Ramadan.

## Ethics Statement

The study was conducted in accordance with the Code of Ethics for human experimentation, the Declaration of Helsinki (World Medical Association, 2013), and was approved by the Research Ethics Committee of Habib Bourguiba University Hospital, Sfax, Tunisia (CCP N°: 1773).

## Author Contributions

MBouz, A-EA, MBouc, and HC designed the experiments. MBouz, A-EA, KG, WD, FE, and HC collected and analyzed the data. Data interpretation and manuscript preparation were undertaken by MBouz and FE. All authors read and approved the final manuscript.

### Conflict of Interest

The authors declare that the research was conducted in the absence of any commercial or financial relationships that could be construed as a potential conflict of interest.
